# A Framework for Prediction of Response to HCV Therapy Using Different Data Mining Techniques

**DOI:** 10.1155/2014/181056

**Published:** 2014-12-11

**Authors:** Enas M. F. El Houby

**Affiliations:** Engineering Division, Systems & Information Department, National Research Centre, El Buhouth Street, Dokki, Cairo 12311, Egypt

## Abstract

Hepatitis C which is a widely spread disease all over the world is a fatal liver disease caused by Hepatitis C Virus (HCV). The only approved therapy is interferon plus ribavirin. The number of responders to this treatment is low, while its cost is high and side effects are undesirable. Treatment response prediction will help in reducing the patients who suffer from the side effects and high costs without achieving recovery. The aim of this research is to develop a framework which can select the best model to predict HCV patients' response to the treatment of HCV from clinical information. The framework contains three phases which are preprocessing phase to prepare the data for applying Data Mining (DM) techniques, DM phase to apply different DM techniques, and evaluation phase to evaluate and compare the performance of the built models and select the best model as the recommended one. Different DM techniques had been applied which are associative classification, artificial neural network, and decision tree to evaluate the framework. The experimental results showed the effectiveness of the framework in selecting the best model which is the model built by associative classification using histology activity index, fibrosis stage, and alanine amino transferase.

## 1. Introduction

Hepatitis C is a contagious liver disease that results from infection with the hepatitis C virus. It can range in severity from a mild illness lasting a few weeks to a serious, lifelong illness. The hepatitis C virus is usually spread when blood from an infected person enters the body of a susceptible person. It is among the most common viruses that infect the liver. Every year, 3-4 million people are infected with the hepatitis C virus. About 150 million people are chronically infected and are at risk of developing liver cirrhosis and/or liver cancer. More than 350 000 people die from hepatitis C-related liver diseases every year. Countries with high rates of chronic infection are Egypt (15%), Pakistan (4.8%), and China (3.2%). There are 6 genotypes of hepatitis C and they may respond differently to treatment. Combination of interferon and ribavirin has been the mainstay of hepatitis C treatment. Unfortunately, interferon is not widely available globally, it is not always well tolerated, some virus genotypes respond better to interferon than others, and many people who take interferon do not finish their treatment. This means that while hepatitis C is generally considered to be a curable disease for many people this is not a reality [1].

Data Mining (DM) is defined as “the extraction of hidden predictive information from large databases” [2]. It is the core step of a broader process, called knowledge discovery in databases. This process includes the application of several preprocessing methods aimed at facilitating the application of the DM algorithm and postprocessing methods aimed at refining and improving the discovered knowledge [3]. Classification is an essential task in DM and machine learning research which aims to predict the classes of future data objects. Classifying patients' dataset to predict response for treatment of HCV is an important research area. Associative Classification (AC) [4, 5], Artificial Neural Network (ANN) [6], and Decision Tree (DT) [6–8] had been used to build classification models which predict patients' response to treatment of HCV from clinical information. Also a hybrid rough genetic algorithm [9] had been applied for classifying studied cases for making decision of HCV treatment.

In this research, a framework has been built to reach the highest performance model to predict HCV patient response to treatment from clinical information. The remainder of the paper is organized as follows. In Section 2, materials and methods are introduced. In Section 3, testing the system and the experimental results are conducted, before drawing conclusions and future work in Section 4.

## 2. Materials and Methods

### 2.1. Patients

Data from 200 Egyptian patients with hepatitis C virus genotype 4, who were treated with combined therapy IFN plus RBV for 2 years, was collected at Cairo University Hospital. The number of patients who responded to the treatment is 83 (41.5%), and that of those who did not respond is 117 (58.5%). For each patient there is a record that contains 12 features which are age, gender, Body Mass Index, albumin, Alanine Aminotransferase (ALT), Aspartate Aminotransferase (AST), alpha-fetoprotein, Histology Activity Index (HAI), viral load, genotype, fibrosis stage, and cirrhosis. In addition, patients' response takes either 0 or 1 value, 0 for nonresponder patients and 1 for responder patients.

### 2.2. Framework for Selecting the Best Model to Predict HCV Patient Response to Treatment

**Figure 1:**
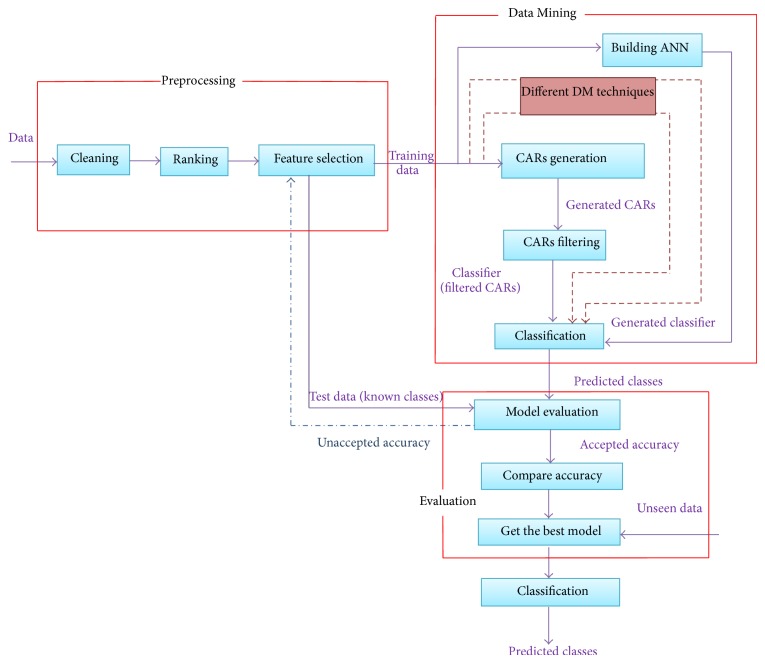
The framework of finding the best model for predicting patients' response to treatment.

In this research, a framework has been built to find the highest performance model that predicts HCV patient response to treatment among various built models using different DM techniques. The framework consists of (1) preprocessing phase to prepare the data for applying data mining techniques and select different combinations of features to build different classifiers; (2) data mining phase to apply different DM techniques; each technique is repeated many times for each time candidate features subset is changed to get the highest possible performance model; (3) evaluation phase to evaluate the performance of all built models using different data mining techniques with different candidate features subsets and by comparing the performance of different models the best model which can predict patient response to treatment is selected. The framework is performing an iterative process; it repeats these three phases until it achieves the best model. The framework including different phases for finding the best model is illustrated in Figure 1.

#### 2.2.1. Data Preprocessing Phase

In preprocessing phase, a series of data preprocessing steps were applied to clean, rank, and select suitable features from patients' data to prepare the data for applying data mining techniques. Features selection is to select a subset of features relevant for the target data mining task from among all the features of the data set. In the filter approach, the features selection method is independent of the data mining algorithm to be applied to the selected features. By contrast, in the wrapper approach the features selection method uses the result of the data mining algorithm to determine how good a given features subset is. The features selection method iteratively generates features subsets (candidate solutions) and evaluates their qualities by the performance of the data mining algorithm applied to that features subset [3].

For ranking the features, the value of each feature importance was calculated as (1 − *P*), where *P* is the value of the corresponding statistical test of association between the candidate features and the target variable which is the patients' response in this case. To calculate the *P* values for different features, the whole data has been used. For categorical features, the *P* values based on Pearson's chi-square were used, while for continuous features the *P* values based on the *F* test were used. The features are sorted by *P* value in ascending order and descending order for (1 − *P*) [10].  Table 1 shows *P* and (1 − *P*) values for categorical features, while Table 2 shows values of continuous features.

In this research, the feature whose (1 − *P*) value is greater than 0.9 is considered, so 9 out of 12 features are considered and the remainder are discarded as it is noticeable in Tables 1 and 2. This selection of these 9 features depends on filter approach. Then wrapper approach is applied for these 9 features, so different combinations of these highest 9 features have been selected in iterative process to select different candidate features subsets, it is simply a selection of different combination of features, and by measuring the performance of the applied DM techniques using these features subsets the suitable subset is selected for each technique.

#### 2.2.2. Data Mining Phase

In DM phase, different DM techniques have been applied to build different classifiers which predict patients' response to HCV treatment using candidate features. Series of steps have been applied to elicit the best model (i.e., the best classifier associated with the set of features which has been used in building this best classifier). These steps can be summarized as follows: for each change in candidate features subset, each DM technique has been conducted *N*-iterations to build *N*-different models for this features subset (where *N* can take any value 1,2, 3,…, *i*) and, in each iteration, different random records (training data) have been selected from the data set to train the classifier. And by measuring the performance of the *N* built models, the best model can be selected for this technique with this candidate features subset. The same process is done for different DM techniques to select the best model for each technique with this candidate features subset. This process is repeated with different candidate features subsets, until it selects the best of all models for each technique. And finally from these best models for different techniques, the best model at all can be selected as the recommended one to predict any future cases of patients' response.

Different DM techniques can be applied using different features subsets to reach the best possible performance model. In this research AC, ANN, and DT have been applied as examples of DM techniques to evaluate the framework. Suppose that the inputs set (patients' features) to the different techniques are selected from *X* = {*X*_1_, *X*_2_, *X*_3_,…, *X*_9_}, where the considered features for features selection in our wrapper approach are 9 as mentioned before in preprocessing phase; from these 9 features, different possible combinations can be taken as input to the DM techniques. And the output to the different techniques is *Y* which represents the result of patient's response to the therapy (0, 1) for nonresponder and responder, respectively. The next subsections give a brief overview of the applied techniques.

(*1) Associative Classification*. AC is a promising classification approach, which has been shown to build more accurate set of rules than traditional classification approaches. AC aims to discover a small set of rules in the database, called Class Association Rules (CARs), to form an accurate classifier. Building a prediction model using associative classification consists of two phases: (1) generate CARs and (2) build a classifier from the generated CARs [11, 12].

CARs generation is done by focusing on a special subset of association rules in which the right-hand side is restricted to the classification class attribute, formally to generate CARs in the patients' response prediction to HCV treatment problem.

Let *D* be the training dataset.

Let *X* be the candidate features subset in *D* used in building the classifier and let *Y* be the set of class labels which is patient response; it takes values (0, 1) for nonresponder and responder.

We say that a data case *d* ∈ *D* contains *I*⊆*X*, a subset of candidate features, if *I*⊆*d*.

A CAR is an implication of the form *I* → *y*, where *I*⊆*X* and *y* ∈ *Y*.

A rule *I* → *y* holds in *D* with* confidence c* if *c*% of cases in *D* that contain *I* is labeled with class *y*.

The rule *I* → *y* has* support s* in *D* if *s*% of the cases in *D* contains *I* and is labeled with class *y*.

CARs generation is done by the adaptation of the existing association rule mining algorithms. In this research the adapted PMA which is used in [5] had been applied to generate a set of CARs. The aim of CARs generation step is to generate CARs which relate patient's response to candidate features subset. So, candidate features subset and the patients' responses are collected together in a database file to be in a form suitable for applying the algorithm. AC algorithms discover frequent rule items (features' values that occur with a class label above the user specified support threshold). A rule item is of the form 〈condset, class〉 where “condset” is a set of features, that is, (feature, value)_*k*_ pair; in this case each feature represents one of the patient's features, where (*k* = 1,…, *i*) according to the number of patients' features in the L.H.S. of the rule, (*i*≤ candidate features number), the class is either (0, 1) for non-responder and responder to the HCV treatment.

The CARs can be generated directly from frequent rule items, where response always represents right-hand side of the rule (class 0 or 1). Examples of generated CAR_3_ (containing 3 features in L.H.S.) and CAR_2_ (containing 2 features in L.H.S.), respectively, are as follows:

(1) HAI,7,fib_stag,2,ALT,1.5 ⟶response,1 support=2Confidence=100%,

(2) {HAI,9},albumin,4.2 ⟶response,0 support=5Confidence=62.5%.

To build classifier from generated CARs most AC algorithms including [11, 12] sort the discovered rules in phase 1 according to their confidence, support values, number of items in the L.H.S. of the rules and prior rule and then apply pruning heuristics to discard redundant and useless rules. A popular pruning method in AC mining called database coverage, which was proposed in [11], had been used for building the classifier. This method tests the set of ranked rules against the training data. For each rule starting with the top ranked rule, the database coverage heuristic tests if the selected rule covers correctly any training data case. In other words, it examines if the selected rule antecedent matches any training data case. If the test turns to be true and both the selected rule and the training data case have a common class, then such a rule is considered a candidate rule in the classifier. If no training data case matches the selected rule or there was a match but no common class was found, then the selected rule will be discarded. The process continues, until there is no training case left or no unselected CARs [11, 12].

The classifier format is 〈CAR_1_, CAR_2_, CAR_3_,…, CAR_*n*_, default  class〉.

(*2) Artificial Neural Network*. Artificial neural network is one of the most widely used machine learning approaches in bioinformatics. ANN is a powerful tool for modeling data, where it is able to capture and represent complex input/output relationships. The true power and advantage of neural networks lie in their ability to represent both linear and nonlinear relationships and in their ability to learn these relationships directly from the data being modeled [13]. ANNs are built from multilayer of nodes linking each other. Typically there are three layers in the network, the input layer, the output layer, and a hidden layer in between them. There are different types of ANNs architectures.

**Figure 2:**
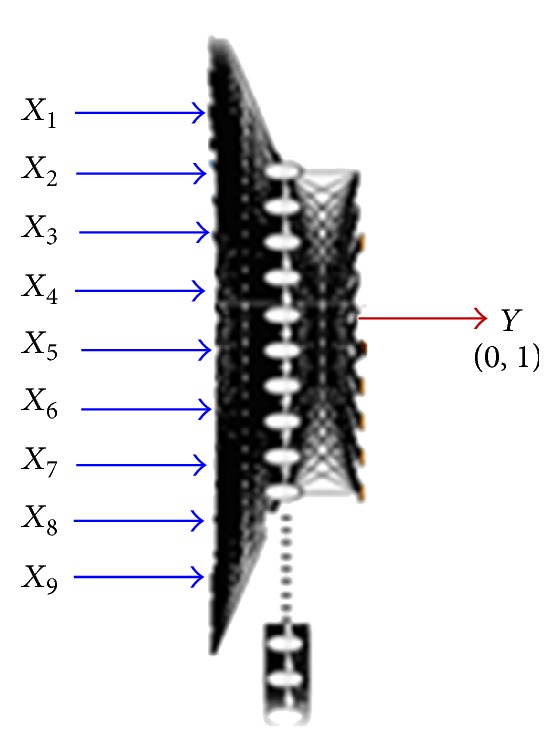
Structure of the built ANN model.

In this research, the back propagation algorithm has been carried out on the model building. The purpose of the built ANN model correctly mapped the input patients' features to the output which is patient's response using training data so that the model can be used to produce the patients' response when the desired output is unknown.  Figure 2 shows the structure of the built model which includes up to 9 neurons of inputs to represent different patients' features subset used in building the model according to the number of candidate features, varied number of neurons for the hidden layer which depends on the used candidate patient features subset and the used training data set, and only one neuron for the output to represent the result of patient's response (0, 1) for nonresponder and responder. Where *X*_1_, *X*_2_, *X*_3_, …, *X*_*n*_ are the inputs (candidate patients' features) with corresponding weights *w*_1_, *w*_2_, *w*_3_,…, *w*_*n*_, *n* can take values up to 9 according to the number of candidate features subsets. The network weights are updated during training in order to improve the network performance. All inputs are multiplied by their corresponding weights and added together to form the net input to the neuron called net. The mathematical expression for net can be written as

(3) net=∑i=1nwiXi+b=w1X1+w2X2+⋯+wnXn+bhhhhhhhhhhhhhhhhhhhwhere  n=1,2,…,9.

The neuron behaves as activation or mapping function *f*(net) to produce an output *Y* which is patient response; it can be expressed as

(4) $$\begin{array}{c} Y=f\left(\text{net}\right)=f\left(\overset{n}{\underset{i=1}{\sum }}w_{i}X_{i}+b\right),\;\left(\text{where}\;n=1,2,\ldots ,9\right), \end{array}$$

where *f* is called the neuron activation function or the neuron transfer function. In this case, the output is limited to two values 0 and 1 depending on the sign of net. The expression of the output *Y* which is patients' response in this case can be written as

(5) $$\begin{array}{c} Y=\left\{\begin{array}{cc} 1 & \text{if}\;\;\text{net}>0\left(\text{reponder}\right),\\ 0 & \text{if}\;\;\text{net}<0\left(\text{Not}\;\text{responder}\right). \end{array}\right. \end{array}$$

(*3) Decision Tree*. Decision tree is one of the widely used data classification techniques because of its simplicity and practical approach. A decision tree is a classifier constructed in a top-down recursive partition divide and conquer manner. In this research, the Classification And Regression Tree (CART) [14] had been used to generate classification tree. CART is widely used statistical procedure based on tree structure that can produce classification and regression trees, depending on whether the dependent variable is categorical or numeric, respectively. It is characterized by the fact that it constructs binary trees; namely, each internal node has exactly two outgoing edges. Classification tree is built through a process known as binary recursive partitioning, which is an iterative process of splitting the data into partitions and then splitting it up further on each of the branches [14].

Since the purpose of this research is to predict the patient response to HCV treatment which can take values (0, 1) for nonresponder and responder, based on input patients' features which are *X*_1_, *X*_2_, *X*_3_, …, *X*_*n*_, *n* can take values up to 9 according to the number of candidate features subsets. So, the results of CART analysis are presented as a decision tree, which is intuitive and which facilitates the allocation of patients into subgroups with respect to the possibility of achieving response or not (1, 0) by following the flowchart form [8].

CART searches for optimal split feature, builds a decision tree structure, and finally classifies all subjects into particular subgroups. During the CART analysis, all newly defined subgroups are investigated at every step of the analysis to determine which feature at what cutoff point yielded the most significant division into two subgroups with respect to estimates response and nonresponse possibilities. The process continues until no more useful splits can be detected.

In this research to partition the data at each stage of tree, a test is performed to select a feature with the lowest entropy. Information Gain (IG) measure is used to select the test feature at each node in the tree. The feature with the highest information gain (greatest entropy reduction) is chosen as a test feature for the current node. This feature minimizes the information needed to classify the samples in the resulting partitions. Such approach minimizes the expected number of tests needed to classify an object [15]. In this research, each node in the tree represents a test on one of the patient's features' values, and the bottom nodes represent the class which takes either 0 or 1 for nonresponder and responder, respectively.

Let *S* be a set of *s* data samples. Since the class has 2 distinct classes for responder and non-responder which are *C*_*i*_  (*i* = 1, 2). Let *s*_*i*_ be the number of samples of *S* in class *C*_*i*_. The expected information needed to classify a sample is given by

(6) $$\begin{array}{c} I\left(s_{1},s_{2}\right)=-\overset{2}{\underset{i=1}{\sum }}P_{i}\;\log_{2}\left(P_{i}\right), \end{array}$$

where *P*_*i*_ is the probability that an arbitrary sample belongs to class *C*_*i*_ and is estimated by *s*_*i*_/*s*.

Let feature *X* have *m* distinct values, {*v*_1_, *v*_2_, …, *v*_*m*_}. Feature *X* can partition sample *S* into *m* subsets {*S*_1_, *S*_2_, …, *S*_*m*_}, where *S*_*j*_ contains those samples in *S* that have value *v*_*j*_. Let *s*_*ij*_ be the number of samples of class *C*_*i*_ in subset *S*_*j*_. The expected information needed to classify a given sample according to feature *X* is given by

(7) EX=∑j=1ms1j+s2jsIs1j,s2j=∑j=1ms1j+s2js−∑i=12Pij log⁡2Pij,

(8) $$\begin{array}{c} \text{Information}\;\text{Gain}\;\left(X\right)=I\left(s_{1},s_{2}\right)-E\left(X\right). \end{array}$$

By substitution from (6), (7) in (8), (9) can be used to get information gain if patient's feature (*X*) has been used to partition the current node:

(9) Information  Gain  X =−∑i=12Pi log⁡2Pi  −∑j=1ms1j+s2js−∑i=12Pijlog⁡2Pij.

#### 2.2.3. Evaluation Phase

In evaluation phase, all the models built using different DM techniques for various candidate features subsets have been evaluated using test dataset of 50 cases which have been selected randomly in each iteration. This dataset is independent of the model building dataset (i.e., training dataset). According to evaluation results, the highest performance model can be selected. The following steps have been followed: for each candidate features subset, among the *N* built models for each technique, the highest performance model has been elicited to represent the best model for this DM technique with this candidate features subset. And by repeating that with different candidate features subsets, we can get the best of all models for each technique with associated features which achieve that best model. By comparing the best selected model for each technique, the highest performance model at all among all techniques has been elicited as the recommended model which can be used to predict future unseen data.

Different statistical information has been used to evaluate the built classifiers. It includes True Positives (TP), False Positives (FP), True Negatives (TN), and False Negatives (FP) together with six performance measures which are* sensitivity* which is the probability that a test result is positive when the disease is present;* specificity* which is the probability that a test result is negative when the disease is not present;* positive predictive value* which is the probability that a disease is present when the result is positive;* negative predictive value* which is the probability that a disease is not present when the result is negative;* Accuracy* which is the probability that the test result conforms to actual value (positive/negative); and also* Area Under Curve (AUC)* which has been used to evaluate the performance; MedCalc has been used to perform ROC curve analysis and calculate AUC, sensitivity, and specificity automatically besides calculating sensitivity and specificity mathematically.

The pseudocode depicted in Algorithm 1 sums up the steps for building different models and finding the best model of all built models to predict patients' response to HCV treatment. As it is shown in Algorithm 1, for each time candidate features subset has been changed (start in step (1) and end in step (10)), steps (2)–(9) have been done to apply each DM technique *N*-times with this change in features subset. In each of the *N*-times for each technique, in steps (4)–(6) data has been selected randomly to build the model using the applied DM technique and the built model has been evaluated using test data. After applying each technique *N*-times (steps (3)–(7)), the best model has been selected in step (8) from the *N* built models for the applied technique. Steps (2)–(9) have been repeated for each technique and ended by getting the best model for that technique with candidate features subset which is selected in step (1). The same process has been repeated (from step (1) to (10)) with each candidate feature subset, and the best model has been selected for each applied technique with each candidate features subset in step (8). After trying all candidate feature subsets, the highest performance model has been selected for each technique associated with the features subset which is used in building this model in step (11). After comparing the highest performance model among the techniques in step (12), the highest performance model at all has been selected among all techniques in step (13).

## 3. Experimental Results

This section shows an empirical performance evaluation of the proposed framework using the applied DM techniques which are ANN, AC, and DT. Data from 200 Egyptian patients with hepatitis C virus who were treated with combined therapy IFN plus RBV for 2 years were used. Extensive experimental studies had been tried in order to get the highest possible performance model; with each change in candidate features subset, *N*-iterations have been tried to build *N*-different models for each of the applied techniques. In this research *N* had been selected to be 6, so for each selection of new candidate features subset 6 different classifiers had been built for each of ANN, AC, and DT. In each iteration, a set of 50 records had been selected randomly out of 200 records to test the model and the remaining 150 records had been used to build the classifiers.

After trying different candidate features subsets and building 6 different classifiers using the various techniques for each change in candidate features subset, we found that for associative classification the subset of features which includes Histology Activity Index (HAI), fibrosis stage, and Alanine Aminotransferase (ALT) is close to the subset of fibrosis stage, Alanine Aminotransferase (ALT), and Alfa-fetoprotein and both subsets give the highest performance for AC; the first subset is selected to build AC model. On the other hand, ANN gives the highest performance using a subset of 5 features instead of 3 for AC, which are Histology Activity Index (HAI), fibrosis stage, viral load, Alpha-fetoprotein, and albumin. For DT Histology Activity Index (HAI), fibrosis stage and Alanine Aminotransferase (ALT) give the highest performance.

After applying different models a great deal of statistical information was supplied; these performance measures had been used to evaluate different classification models as shown in Tables 3, 4, and 5. These tables show the 6 iterations for each technique with selected features subset which gives the highest performance for that technique. The ANN models have sensitivity ranging from 76% to 92% in average 87.33% and specificity ranging from 20% to 80% in average 50.66%, while the AC models have sensitivity values ranging from 45.5% to 81.8% in average 62.58% and specificity ranging from 89.3% to 100% in average 93.47% and DT models have sensitivity ranging from 54.5% to 88.9% in average 68.53% and specificity from 71.1% to 77.5% in average 74.38%. For the positive predictive values, the values diverse from 53.5% to 79.2% for ANN in average 65.28%, from 76.9% to 100% in average 84.95% for AC, and from 35.3% to 55.6% in average 44.27% for DT. Concerning the negative predictive values, they vary from 57% to 84% in average 75.38% for ANN, from 67.6% to 88.6% in average 79.87% for AC, and from 81.25 to 97.0% in average 89.16% for DT. AUC values vary from 54.5% to 77% in average 67.95% for ANN, while for AC they vary from 67.4% to 90.9% in average 78.05% and from 62.8% to 83.2% in average 71.95% for DT. The diagnostic accuracy for ANN changes from 56% to 78% in average 69%, while for AC it changes from 70% to 92% in average 81.33% and for DT it changes from 68% to 80% in average 73.33%. By comparing all performance measures for different models of ANNs, ACs, and DTs which are shown in Tables 3, 4, and 5, it is clear that the best model built using AC outperforms that of ANN and DT.

The structures of different constructed models have been changed with the change of the candidate features subsets and training datasets used in constructing them. For ANNs, the numbers of neurons in hidden layer were varied for different models as shown in Table 3. For ACs, the numbers of generated CARs and the numbers of filtered rules which represent the classifiers were varied as shown in Table 4. And for DTs the numbers of nodes and the numbers of pruning levels were varied as shown in Table 5. As it is shown in Table 3, from ANN1 to ANN5 with increasing the numbers of neurons in hidden layer, the ANN performance measures increase, but that does not apply to ANN6; although it owns the least number of neurons in hidden layer, it gives the best performance, so there are no clear criteria depending on the number of neurons in hidden layer which can be used in optimizing the model. For ACs in Table 4, although the numbers of generated CARs and the numbers of filtered rules are close to each other from AC1 to AC6, there is significant difference in the performance measures of these models. It is the same for DT as shown in Table 5; the numbers of nodes and the numbers of pruning levels are close to each other from DT1 to DT6; however, there is clear difference in the performance measures of these models. Since there are no criteria depending on the structures of constructed models which can be used to optimize these models, the major concern was to optimize the performance measures rather than the structure parameters.

**Figure 3:**
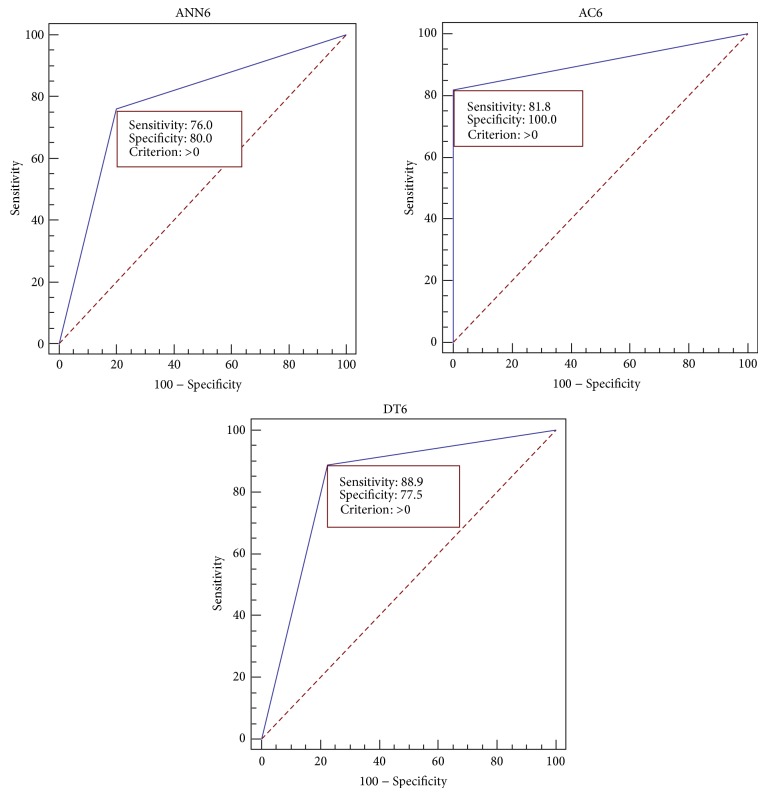
The ROC curves for the best models of ANN, AC, and DT with sensitivity and specificity values at the optimal cutoff points.

**Figure 4:**
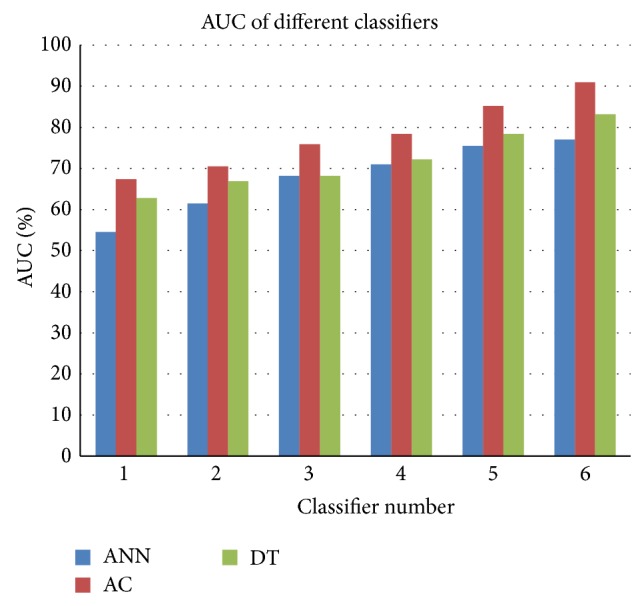
Comparison of AUC for different ANNs, ACs, and DTs.

**Figure 5:**
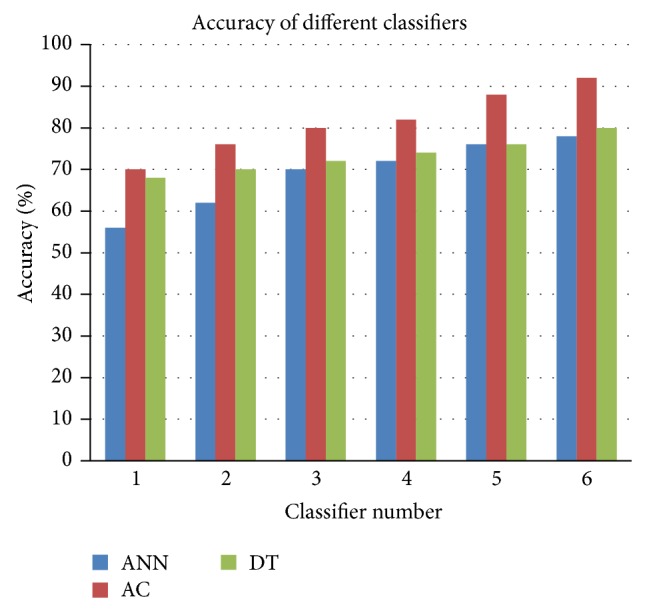
Comparison of accuracy for different ANNs, ACs, and DTs.

Since we should select the highest performance model to use it as a recommended model for future prediction, we should focus on the individual model's performance not the average performance. By focusing on the best model of each of the three techniques which are AC6, ANN6, and DT6 as indicated in Table 6, it is shown that the best accuracy of AC is 92% while for ANN it is 78% and it is 80% for DT. Comparing the Receiver Operating Characteristic (ROC) curves for ANN6, AC6, and DT6 models with their sensitivity and specificity values at the optimal cutoff points as shown in Figure 3, it is clear that AC6 is the closest to the top left of ROC curves and it satisfies the highest AUC. This means that the AC6 has the highest value of accuracy, although the sensitivity of DT6 is higher than that of AC6, but still the specificity of AC6 is higher and so are the accuracy and AUC.  Figure 4 shows the comparison of Area Under Curve (AUC) for different ANNs, ACs, and DTs, while Figure 5 shows the comparison of accuracy for different ANNs, ACs, and DTs.

By comparing all performance measures for different ACs, DTs, and ANNs, it is clear that the best model built using AC outperforms that of DT and ANN, although AC needs 3 features while ANN needs 5 features but DT still needs 3 features to give the best performance. Based on our results, we recommend the best model built using AC with features subset including Histology Activity Index (HAI), fibrosis stage, and Alanine Aminotransferase (ALT) for the prediction of responders to HCV treatments.

## 4. Conclusion and Future Work

In this research, a framework has been developed to compare different data mining techniques' performance in predicting patients' response to treatment of HCV from clinical information. Three data mining techniques which are ANN, AC, and DT have been applied for the prediction. The subset of features suitable for each of the three techniques has been selected to reach the highest possible performance. Then the comparison among the three best models for different techniques has been conducted. 200 patients treated with IFN and RBV were analyzed and used to evaluate the three techniques. The experiment results showed that the three techniques give acceptable results; the best model built using AC outperforms that of ANN and DT although AC needs 3 features while ANN needs 5 features but DT still needs 3 features to give the best performance. The results showed that as sensitivity and specificity increase the AUC increases and so does the accuracy. The best accuracy for the AC is 92% while for ANN it is 78% and it is 80% for DT.

In the future, we hope that we have more available data set to train the classifiers and try more experiments and more analyses to the data. Also we hope to try many other techniques and combine more than one technique to reach as high accuracy as possible.

## Figures and Tables

**Algorithm 1:**
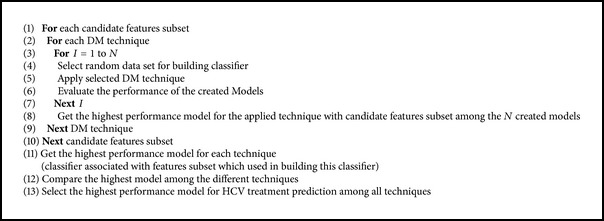
The pseudocode of finding the best model for predicting patients' response to HCV treatment.

**Table 1:** Chi-square test for categorical features.

Features	*P* value	(1 − *P*)
Fibrosis stage	0.001	0.999
Histology Activity Index (HAI)	0.001	0.999
Cirrhosis	0.03	0.97
Age	0.17	0.83
Genotype	0.362	0.638
Gender	0.75	0.25

**Table 2:** *F* test for continuous features.

Features	*P* value	(1 − *P*)
Alanine Aminotransferase (ALT)	<0.0001	>0.999
Viral load	<0.0001	>0.999
Body Mass Index (BMI)	<0.0001	>0.999
Albumin	<0.0001	>0.999
Alpha-fetoprotein	<0.007	>0.993
Aspartate Aminotransferase (AST)	<0.02	>0.98

**Table 3:** Performances of 6 iterations of ANN with selected features.

ANN number	Hidden layer neurons	TP	TN	Positive predictive value %	Negative predictive value %	Sensitivity %	Specificity %	AUC %	Accuracy %
ANN1	70	23	5	53.5	71.4	92	20	54.5	56
ANN2	90	23	8	57	57	92	32	61.5	62
ANN3	125	22	13	65	81	88	52	68.2	70
ANN4	150	22	14	66	82	88	56	71.0	72
ANN5	180	22	16	71	84	88	64	75.5	76
ANN6	43	19	20	79.2	76.9	76	80	77.0	78

Average		22	13	65.28	75.38	87.33	50.66	67.95	69

**Table 4:** Performances of 6 iterations of AC with selected features.

AC number	Generated rules	Filtered rules	TP	TN	Positive predictive value %	Negative predictive value %	Sensitivity %	Specificity %	AUC %	Accuracy %
AC1	116	56	10	25	76.9	67.6	45.5	89.3	67.4	70
AC2	119	58	9	29	81.8	74.4	47.4	93.5	70.5	76
AC3	113	60	11	29	78.6	80.5	61.1	90.6	75.9	80
AC4	112	59	12	29	85.7	80.6	63.2	93.5	78.4	82
AC5	118	60	13	31	86.7	88.6	76.5	93.9	85.2	88
AC6	119	61	18	28	100	87.5	81.8	100	90.9	92

Average			12	29	84.95	79.87	62.58	93.47	78.05	81.33

**Table 5:** Performances of 6 iterations of DT with selected features.

DT number	Number of nodes	Number of pruning levels	TP	TN	Positive predictive value %	Negative predictive value %	Sensitivity %	Specificity %	AUC %	Accuracy %
DT1	45	11	7	27	35.3	84.8	54.5	71.1	62.8	68
DT2	51	9	7	28	38.9	87.5	60	71.8	66.9	70
DT3	45	13	10	26	55.6	81.25	60	76.5	68.2	72
DT4	53	17	8	28	44.4	90.6	70	74.4	72.2	74
DT5	47	11	8	30	44.4	93.8	77.8	75	78.4	76
DT6	61	11	8	32	47.0	97.0	88.9	77.5	83.2	80

Average			8	29	44.27	89.16	68.53	74.38	71.95	73.33

**Table 6:** Performance of the best AC, ANN, and DT.

Classifier number	TP	TN	Positive predictive value %	Negative predictive value %	Sensitivity %	Specificity %	AUC %	Accuracy %
AC6	18	28	100	87.5	81.8	100	90.9	92
ANN6	19	20	79.2	76.9	76	80	77.0	78
DT6	8	32	47.0	97.0	88.9	77.5	83.2	80
